# Rapid, modular and reliable construction of complex mammalian gene circuits

**DOI:** 10.1093/nar/gkt605

**Published:** 2013-07-11

**Authors:** Patrick Guye, Yinqing Li, Liliana Wroblewska, Xavier Duportet, Ron Weiss

**Affiliations:** ^1^Department of Biological Engineering, Massachusetts Institute of Technology, 40 Ames Street, Cambridge, MA 02142, USA, ^2^Department of Electrical Engineering and Computer Science, Massachusetts Institute of Technology, 40 Ames Street, Cambridge, MA 02142, USA and ^3^INRIA Paris-Rocquencourt, Le Chesnay, 78153, France

## Abstract

We developed a framework for quick and reliable construction of complex gene circuits for genetically engineering mammalian cells. Our hierarchical framework is based on a novel nucleotide addressing system for defining the position of each part in an overall circuit. With this framework, we demonstrate construction of synthetic gene circuits of up to 64 kb in size comprising 11 transcription units and 33 basic parts. We show robust gene expression control of multiple transcription units by small molecule inducers in human cells with transient transfection and stable chromosomal integration of these circuits. This framework enables development of complex gene circuits for engineering mammalian cells with unprecedented speed, reliability and scalability and should have broad applicability in a variety of areas including mammalian cell fermentation, cell fate reprogramming and cell-based assays.

## INTRODUCTION

The ability to introduce into mammalian cells genetic circuits that contain multiple transcription units (TUs) is of great interest for a variety of applications including biotechnology (1), gene therapy (2), systems/synthetic biology (3) and reprogramming cell fate and functions (4), as well as basic biological research (5). Encoding such multi-TU gene circuits on single vectors offers several advantages over using separate vectors (6,7), for example, to improve correlation in gene expression between the different circuit elements and for an integration of the entire circuit into a single genomic locus. However, the construction of such large single-vector circuits is challenging because of long and/or repetitive sequences and the need for genetic elements that impart robust expression in mammalian cells. Existing DNA assembly methods are often not well suited for manipulating large collections of mammalian sequences. For example, methods that rely on the use of Type IIs restriction enzymes (8) can be problematic because these restriction sites occur frequently in mammalian promoters and genes. Other methods require multiple rounds of cloning (6,9), cloning in yeast (10) or polymerase chain reaction (PCR) (7). With PCR, the precision of even high-fidelity polymerases is insufficient for reliable and error-free large-scale amplification (Supplementary Table S1). Furthermore, multi-TU gene circuits lacking insulating elements suffer from transcriptional interference and are significantly hampered in their function (11). To address these issues, we developed a new framework for quick and reliable assembly of functional complex mammalian gene circuits. Here, we describe in detail the components, steps and mechanisms underlying the framework. We demonstrate efficient and robust construction of circuits with various sizes and number of assembled parts, and show that assembly works well despite repetitive sequences present in some of the parts. The resulting gene circuits were functionally assessed in transfection as well as stable genomic integration and behaved according to their predicted phenotypes. The framework described here can also prove to be valuable for building large-scale mammalian genetic module libraries, and is well suited for generation of stable cell lines with multielement circuits.

## MATERIALS AND METHODS

### Strains

*Escherichia coli* 10G (Lucigen Corp., Middletown, WI, USA) were used for most cloning steps except for the pJazz-based and the bacterial artificial chromosome (BAC)-based carrier vectors. pJazz/BigEasy v2.0 Linear Cloning System and pSMART-BAC (Lucigen Corp.) were purchased and used according to their manual in their respective strains (BigEasy TSA and BAC-Optimized Replicator v2.0). Antibiotic was used with following concentrations: 100 µg/ml Ampicillin, 50 µg/ml Kanamycin, 25 µg/ml Chloramphenicol. Gel extraction was done with the Qiagen QIAquick Gel Extraction Kit. PCR purification was done using the Qiagen QIAquick PCR Purification Kit. Miniprepping of DNA was done using the Qiagen Qiaprep Spin Miniprep Kit. Some minipreps were automated on a Qiagen Qiacube using the same miniprep kit.

### Library of genes and promoters

The Gateway BP reaction was performed according to the manufacturer's manual (Life technologies, Carlsbad, CA, USA). Briefly, to create the promoter library, the promoter sequences of interest were amplified, digested and inserted into pENTR_L4_R1 that was cut with compatible restriction enzymes. To create the gene library, the gene of interest was amplified with attB1-site in the forward primer and attB2-site in the reverse primer. Ten femtomoles of the PCR product was mixed with 5 fmol of pDONR221P1P2 and incubated with 1 µl of BP clonase II (11789-020, Life technologies) for 1 h. One microliter of the reaction was transformed into ccdB sensitive competent *E. coli* cells. Alternatively, the gene sequences of interest were amplified, digested and inserted into pENTR_L1_L2 cut with compatible restriction enzymes.

### Gateway LR reaction

Gateway LR reactions were performed according to the manufacturer's manual (Life technologies, Carlsbad). Briefly, 5 fmol of each of pENTR_L4_R1, pENTR_L1_L2 and pZDonor_Seq(n)-GTW-Seq(*n* + 1) that contains Gateway cassette of pDEST_R4_R2 were mixed and incubated with 1 µl of LR clonase II mix (11971-020, Life technologies) for 16 h. One microliter of the reaction was transformed into ccdB sensitive competent *E. coli* cells.

### Digestion of vectors containing the basic assembly units

Seventy femtomoles of each vector containing a basic assembly unit were pooled and digested in a total volume of 20 µl for 6 h using 10 U of the restriction enzyme I-SceI (R0694, NEB Biolabs, MA, USA). Subsequently, this digest was purified using the QIAGEN Qiaquick PCR purification kit and eluted in 30 µl of warm Tris-EDTA (TE) buffer. Alternatively, the I-SceI digest was heat-inactivated (65°C, 20 min) and used without further purification.

### Digestion of the adaptor vector

Two hundred eighty femtomoles of the adaptor vector required for proper circuit closure was digested in a total volume of 20 µl with 10 U each of restriction enzymes XbaI and XhoI (R0146, R0145, NEB Biolabs) for 3 h, purified using the QIAGEN Qiaquick PCR purification kit and eluted in 30 µl of warm TE buffer. Alternatively, the digest was heat-inactivated (65°C, 20 min) and used without further purification.

### Digestion of the carrier vector

One hundred forty femtomoles of the carrier vector was digested in a total volume of 20 µl with 4 U of the restriction enzyme FseI or PacI (R0588, R0547 NEB Biolabs) for 3 h, purified using the QIAGEN Qiaquick PCR purification kit and eluted in 30 µl of warm TE buffer. Alternatively, the digest was heat-inactivated (65°C, 20 min) and used without further purification.

### One-step assembly using exonuclease, ligase and polymerase (Gibson Reaction)

The reaction buffer was made according to Gibson's original recipe (12). Briefly, isothermal reaction buffer (IRB) was prepared on ice and stored at −20°C: 25% PEG-8000, 500 mM Tris–HCl, pH 7.5, 50 mM MgCl_2_, 50 mM dithiothreitol (DTT), 1 mM each of dATP, dCTP, dGTP and dTTP and 5 mM NAD. Assembly Master Mix was prepared on ice and stored at −20°C: 320 μl IRB buffer, 0.64 μl of 10 U/μl T5 exonuclease (T5E4111K, Epicentre Biotechnologies, WI, USA), 20 μl of 2 U/μl Phusion polymerase (F-530, NEB Biolabs), 160 μl of 40 U/μl Taq ligase (M0208, NEB Biolabs), deionized water to a 1.2 ml total volume. Seven femtomoles of each part (digested adaptor vector, digested carrier vector and digested pool of assembly units) were combined in a 200 µl PCR reaction tube on ice and filled up to a 5 µl of total volume with deionized water. The mix was then added to 15 µl of the Assembly Master Mix, and the reaction was incubated at 50°C for 1 h. This reaction (2–5 µl) was then transformed into competent *E. coli* cells.

### Hierarchical assembly

The assembly units and the adaptor were assembled into the hierarchical pJazz carrier vector. One hundred forty femtomoles of assembled vector was digested in total volume of 20 µl for 6 h using 10 U of the restriction enzyme I-SceI. Seven femtomoles of this digest was combined with 7 fmol of each additional part (digested adaptor vector, digested carrier vector and digested pool of assembly units). Kanamycin resistance adaptor and Tetracycline resistance adaptors were used in an alternating manner. A one-step assembly protocol was applied on this mixture.

### Cell culture

HEK293FT (Invitrogen, Carlsbad, CA, USA) cells were cultured in supplemented Dulbecco’s modified Eagle’s medium according to their manual. Chemical DNA transfection was performed using Qiagen SuperFect Transfection Reagent (QIAGEN, Hilden, Germany) or Metafectene Pro (Biontex, Martinsried, Germany). In brief, 800 000 cells were seeded into a 10-cm^2^ well and immediately transfected with 2 µg of DNA. The medium was replaced 6 h after transfection. To induce the Tet-On system, Doxycycline (Clontech, Mountain View, CA, USA) was added to culture media at 1 µg/ml. To induce the Rheo system, Genostat ligand (EMD Millipore, Burlington, MA, USA) was added to the cell culture at a final concentration of 5 nM. Targeted integration into the AAVS1 locus: The carrier vector was stably integrated (13) into the chromosome of HEK293 cells and stable clones were selected with 1 μg/ml of Puromycin (Xavier Duportet *et al.*, submitted for publication).

### Microscope imaging

Images were taken using a Leica TCS SP5 II 405UV confocal microscope (Leica Microsystems, Bannockburn, IL, USA). Images were acquired using a sequential scan. First scan: excitation/laser lines: 488 nm, emission: 495–539 nm; second scan: excitation/laser lines: 458 and 543 nm, emission: 462–487 and 547–800 nm, respectively.

### Flow cytometry measurement

Flow cytometry measurement was carried out on BD LSR II in Koch Institute Flow Cytometry Core at MIT. Data were collected in BD FACSDiva software and analyzed in Flowjo (Tree Star, Inc. Ashland, OR, USA)

### Algorithms

The algorithm and parameters for designing the unique oligonucleotide sequences are detailed in Supplementary Method.

## RESULTS

### Genetic circuit design and construction

**Figure 1.**
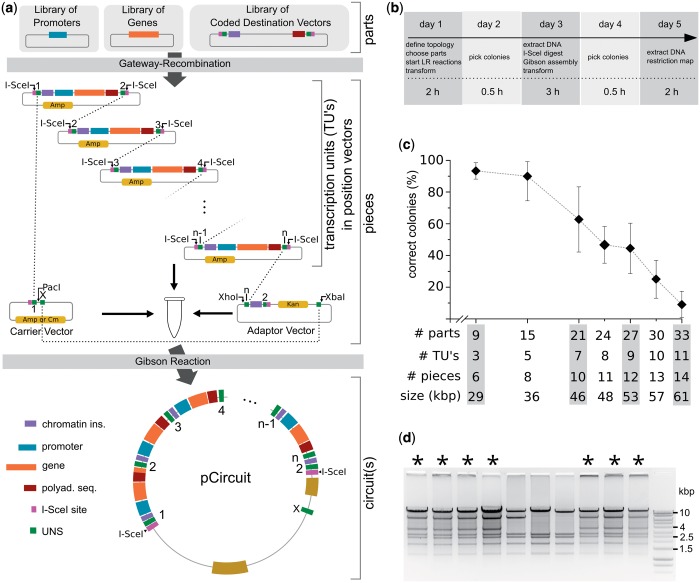
Overview of the DNA assembly process and its efficiency. (**a**) Schematics of the assembly process. For every TU, the chosen promoter, gene and destination vector are recombined using the Gateway LR reaction. The UNS in the destination vector determines the TU’s position within the gene circuit. The resulting position vectors are pooled, digested with I-SceI and combined with equimolar amounts of predigested carrier vector and adaptor vector. The Gibson reaction mix exposes the UNS, permitting annealing, extension and ligation to form a single large vector (pCircuit). pCircuit itself can be reused for a further round of assemblies by digesting it with I-SceI, freeing the 5-TU circuit flanked by the UNS 1 and 2. (**b**) Timeline and steps for circuit assembly starting from libraries of parts. If all TUs are already available, the assembly process starts from day 3 and takes 2 days to finish. The approximate hours of benchwork needed each day is indicated in the bottom row (h = hours). (**c**) TUs were assembled into a linear carrier vector (customized pJazz) (14). The percentage reflects the number of colonies containing the correct vector vs. the number of analyzed colonies. Size in kilobases is the average size (multiple different circuits tested per # of parts) (**d**) Accuracy of the assembly process for a 7-TU circuit. Asterisk: correct restriction digest.

Our assembly method integrates multisite Gateway recombination, Gibson assembly (12) and a nucleotide-addressing system for defining the position of every part in the final overall vector (Figure 1a). First, the user chooses promoter/gene pairs from a sequence-verified library of parts and then determines the circuit position of each of these TUs by Gateway recombination with an appropriate customized Gateway destination vectors. The resulting vectors, called position vectors, contain nucleotide sequences that specify the position of each TU in the final circuit vector. Position vectors are verified by restriction mapping (>90% usually correct) (15), and then digested and assembled together with a carrier vector and an adaptor vector using a Gibson reaction to form the final vector. Our customized Gateway destination vectors contain (from 5′ to 3′) an I-SceI restriction site, a unique nucleotide sequence (UNS), a tandem repeat of the core cHS4 chromatin insulator (16), a Gateway recombination cassette, a polyadenylation sequence, another UNS and another I-SceI restriction site (Figure 1a, Supplementary Figure S1a). The I-SceI recognition sites and UNSs form the core of the nucleotide-addressing system. Digesting the position vector with I-SceI releases the TU flanked by the two UNSs. The TU should not contain the 18-bp I-SceI site, but the likelihood of this sequence being present in a TU is small—neither the mouse genome nor the human genomes contain this recognition site. UNSs comprise a series of 40-bp nucleotide sequences that are designed using a computational algorithm to maximize the probability of annealing to the complementary UNS during the Gibson reaction and to minimize hairpin formation when exposed as single-stranded DNA (Supplementary Figure S1b, algorithm described in Supplementary Methods). Chromatin insulator and polyadenylation sequences are included in each position vector for robust mammalian gene expression. Once assembled, chromatin insulators from adjacent assembled transcriptional units form insulation pairs that are used to dampen crosstalk by transcriptional regulators (11) as well as the spreading of genomic silencing (17). The carrier vector contains sequences necessary for propagation in *E. coli* as well as UNS 1 and X, where UNS X is used to link the last TU in the circuit to the carrier vector. Additional genetic elements, for instance episomal sequences or genomic recombination sites, can be added to the carrier vector to obtain other desired functionality. To link the last position vector to the carrier vector at UNS X, an adaptor vector is chosen from an adaptor library. The adaptor also provides a second selection marker (e.g. Kanamycin or Tetracycline) to select against empty vector backbones during Gibson assembly. For example, a 5-TU gene circuit can be assembled from position vectors with UNS pairs 1–2, 2–3, 3–4, 4–5 and 5–6 and an adaptor vector containing UNS 6–X (Figure 1a). A list of components available as part of the platform is available in Supplementary Table S2. To facilitate gel electrophoresis analysis of the large gene circuits, we developed an algorithm to create high-resolution restriction maps (Supplementary Figure S2, restriction map algorithm described in Supplementary Method S2).

### Assembly method efficiency

We demonstrate the efficiency of our method by assembling >30 basic parts into single vectors (Figure 1c and d and Supplementary Figure S2). The entire construction process from basic parts (promoters, genes) to a transfection- or integration-ready circuit vector requires <5 days with a workload of a few hours a day (Figure 1b). Starting from preexisting TUs, construction takes only 2 days. Figure 1c shows the percentage of bacterial colonies that contained the correct circuits for assemblies of up to 33 basic parts and a final vector size of 64 kb. Our data suggest that for assemblies with <21 parts or 7 TUs, it is usually sufficient to screen only two colonies (<10% probability of both colonies not containing the correct circuit, Figure 1b).

### Reliable, robust expression and exogenous control

**Figure 2.**
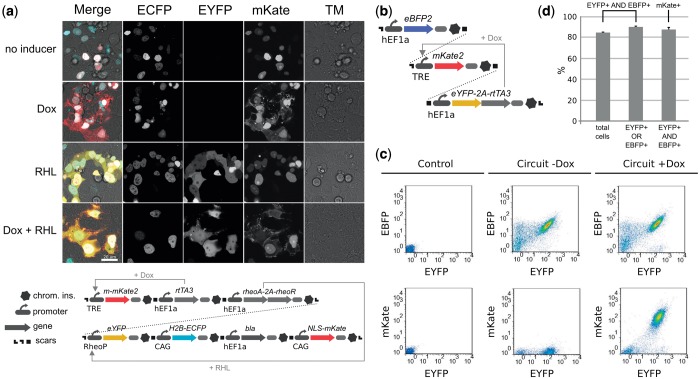
Gene circuit function in mammalian cells. (**a**) Microscope images from transfection of a 7-TU, 39-kb gene circuit. The circuit constitutively expresses nuclear-localized ECFP, nuclear-localized mKate, rtTA3, RheoAct/RheoRec and Blasticidin. On addition of Doxycycline, expression of membrane-localized mKate2 is induced. EYFP is expressed on addition of Genostat ligand. Chrom. ins., chromatin insulator. Scale bar: 20 µm (**b**) A 3-TU, 17-kb gene circuit that was stably integrated into HEK293FT cells. (**c**) Flow cytometry dot-plots for cells not containing the circuit (Control), and cells containing the chromosomally integrated circuit with or without Doxycycline (±Dox). (**d**) Assessing the functionality of the gene circuit described in (b). Percentage of cells expressing EYFP and EBFP in (first column) total population, (second column) EYFP or EBFP expressing population and (third column) percentage of cells expressing mKate in EYFP and EBFP double-positive cells on addition of Doxycycline.

We assayed the behavior in mammalian cells of large gene circuits constructed with our method. A 39-kb vector containing a 7-TU circuit that was assembled into a BAC carrier vector (Figure 2a and restriction digest/analysis in Supplementary Figure S2), exhibited robust gene expression on induction with Doxycycline and/or Rheo ligand when transfected into HEK293FT cells (Figure 2a). We also compared gene expression from a single transfected plasmid to gene expression from three co-transfected plasmids that contain the same circuit elements. The circuit comprises constitutive expression of Enhanced Blue Fluorescent Protein (EBFP), Enhanced Yellow Fluorescent Protein (EYFP) and a reverse tetracycline transactivator (rtTA3) and Doxycycline-inducible mKate (Figure 2b). EYFP serves as a transfection marker. The results show that having the entire circuit in a single plasmid significantly reduces EBFP and mKate variance over a wide range of transfection levels, based on flow cytometry analysis (Supplementary Figure S3). To determine if circuits assembled with this framework perform well with stable chromosomal integration, we integrated the above 3-TU circuit into the AAVS1 locus of HEK293FT cells and quantified the resulting fluorescence. After 17 days, >85% of polyclonal cells co-expressed EBFP and EYFP with similar levels of expression and 87% of these EBFP-EYFP double-positive cells expressed mKate in an inducible manner (Figure 2c and d).

### Hierarchical circuit assembly

Our method supports hierarchical assembly of gene circuits (Supplementary Figure S4). Hierarchical construction and reuse of genetic modular circuits should facilitate design and implementation of high-order gene circuits and larger systems (18). With this approach to the creation of sophisticated systems, one can first assemble and verify simpler modules (e.g. bistable switches, cascades, biosynthesis pathways, etc.) and also obtain such modules from other sources, before proceeding to the construction of the complete systems. Recently, a plug-and-play method (19) based on restriction enzymes was developed that supports reuse and repurposing of existing genetic circuits for the construction of new bacterial synthetic gene networks. In our framework, each assembled circuit vector contains an *I-SceI* site upstream of the vector’s 5′ UNS 1 and an *I-SceI* site downstream of UNS 2 at the 3′-end (Figure 1a). Digestion with *I-SceI* creates a position 1–2 vector piece that can be assembled with other position vectors and an appropriate adaptor vector. Two different adaptor vectors, containing either Kanamycin or Tetracycline resistance, are used in an alternating fashion to select against the parental modules. To demonstrate recursive assembly of gene circuits, we first designed and built a 7-TU, 45-kb module. The module contains three inducible promoters and four constitutive promoters expressing inducible regulators, selection markers and fluorescent reporters. After constructing this module, we then used it to create a larger 12-TU circuit (36 parts, 63 kb, Supplementary Figure S4). The additional 5 TUs that were added in the second step include parts that are already present in the 7-TU circuit, allowing us to determine whether our hierarchical assembly method is robust to repetitive sequences, and indeed the construction was successful (Supplementary Figure S4).

## DISCUSSION

The framework described here for assembling complex genetic circuits uses readily available reagents and enzymes and is reliable, efficient, modular and supports a hierarchical construction scheme. We assemble large and complex gene and demonstrate robust regulation of gene expression within these multigene circuits using small molecule inducers in human cells with both transient transfection and stable chromosomal integration. The assembly of repetitive sequences (e.g. multiple repeats of the same promoter or gene) did not result in undesirable recombination events or genetic stability issues (confirmed by sequencing, data not shown). Because our framework does not rely on restriction enzymes, it is highly flexible and can be used for assembly of components constructed using other cloning methods, (e.g. Golden Gate assembly). Also, gene circuit modules with specialized functions can be validated and stored separately, and combined when needed based on hierarchical system design, yielding large circuits with complex phenotypes. We anticipate that our approach will be valuable for building large-scale gene circuit libraries with reliable gene expression, and will be suitable for the generation of stable cell lines with functional multielement circuits. This will greatly benefit the rapidly growing field of mammalian synthetic biology as well as facilitate genetic engineering of mammalian cells with complex multigene circuits. The method described here is not restricted to mammalian cell engineering. By extending the library of parts (genes, promoters) and appropriate modifications of inter-TU regions, the approach can support rapid genetic engineering of many other organisms.

## SUPPLEMENTARY DATA

Supplementary Data are available at NAR Online, including [20–27].

## FUNDING

Defence Advanced Research Projects Agency [HR0011-12-C-0067, DARPA-BAA-11-23]; National Science Foundation [CBET-0939511]; Synthetic Biology Engineering Research Center (SynBERC) [SA5284-11210]; National Institutes of Health [5-R01-CA155320-02]; Swiss National Science Foundation [PA00P3_131492 to P.G.]; the Ernst Schering Foundation (to P.G.). Funding for open access charge: SynBERC [SA5284-11210].

*Conflict of interest statement.* None declared.

## Supplementary Material

Supplementary Data
