# Supplementary material for: Rapid, modular and reliable construction of complex mammalian gene circuits
Source: Nucleic Acids Res. 2013 Jul 11;41(16):e156. doi: 10.1093/nar/gkt605 (PMC3763561; doi:10.1093/nar/gkt605)
Supplement: Supplementary Data [file supp_41_16_e156__index.html]

Rapid, modular and reliable construction of complex mammalian gene circuits — Rapid, modular and reliable construction of complex mammalian gene circuits — Supplementary Data 

# Rapid, modular and reliable construction of complex mammalian gene circuits

## 

files

**Files in this Data Supplement:**

- Supplementary Data - pdf file
- Supplementary Data - pdf file
