# Supplementary material for: Rapid, modular and reliable construction of complex mammalian gene circuits
Source: Nucleic Acids Res. 2013 Jul 11;41(16):e156. doi: 10.1093/nar/gkt605 (PMC3763561; doi:10.1093/nar/gkt605)
Supplement: Supplementary Data [file supp_gkt605_nar-01230-met-k-2013-File004.pdf]

Polymerase Name	Error Rate	Length of PCR template			Reference
		1kbp	10kbp	100kbp	
Taq DNA Polymerase	2.85×10^{-4}	855.00%	8550.00%	85500.00%	1
Taq DNA Polymerase	1×10^{-4}	300.00%	3000.00%	30000.00%	2a
HotStar HiFidelity Polymerase	1×10^{-5}	30.00%	300.00%	3000.00%	2b
Pfx50™ DNA Polymerase	2×10^{-6}	17.10%	171.00%	1710.00%	2c
VELOCITY DNA Polymerase	4.4×10^{-7}	1.32%	13.20%	132.00%	2d
PfuUltra High Fidelity DNA Polymerase	4.3×10^{-7}	1.29%	12.90%	129.00%	2e
Phusion® High Fidelity DNA Polymerase	4.4×10^{-7}	1.32%	13.20%	132.00%	2f

Supplementary Table 1. A comparison of the fidelity of commercial DNA polymerases. The error rates are taken from the providers' documentation / website (see references). The percentages indicate the probability of an amplicon having at least one base-pair mutation, which is the same as the percentage of mutated amplicons in the total amplified products. A value higher than 100% means that there is more than one mutation per amplicon on average. Using a long template (1, 10, 100 kilobases) in PCR leads to an increase in the probability of having mutated amplification products. Note that the percentage only depends on total length of sequence amplified (see derivation below). Splitting the template into smaller pieces for amplification does not reduce the mutation percentage. Even for polymerases that perform best, we estimate that at least 13% of the PCR products contain a mutation for every 10 kilobases. For PCR-amplifying sequence with a total length of 100 kilobases, virtually every product contains at least one mutation.

To estimate the percentage of mutated DNA molecules, let us define

B : total number of base-pairs in the final amplified products

L : length of sequence template

S : number of total PCR cycling

$N(d)$: number of DNA molecules after the d th cycle

λ : error rate, defined as the probability of introducing one base-pair mutation per base-pair extension per molecule per cycle

p : percentage of DNA molecules, which contain at least one base-pair mutation, in the total amplified DNA molecules

The number of DNA molecules after the d th cycle is

– ——

The number of mutated DNA molecules, which come from the mutated DNA after the d th cycle, in the final amplified products is

Summing up mutated DNA molecules that originated from different cycles gives the total number of mutated DNA molecules in the final amplified products. The percentage of

DNA molecules that contain at least one base-pair mutation in the total amplified DNA molecules can be calculated as the number of total amplified DNA molecules divided by the number of mutated DNA molecules, as the following

Polymerase reference

1	Tindall, K.R., and Kunkel, T.A. Fidelity of DNA synthesis by the Thermus aquaticus DNA polymerase. (1988) Biochemistry 27, p6008-6013.
2a	http://www.neb.com/nebecomm/tech_reference/polymerases/properties_dna_polymerases.asp#.UBhsg7TbByo
2b	http://www.qiagen.com/products/pcr/hotstartaqsystem/hotstartaqplusmastermix.aspx?rp=1000966&rpg=0
2c	http://www.invitrogen.com/1/1/1720-pcr-enzyme-selection-kit-high-fidelity.html
2d	http://www.bioline.com/h_prod_detail.asp?itemid=15
2e	http://www.genomics.agilent.com/CollectionSubpage.aspx?PageType=Product&SubPageType=ProductData&PageID=1276
2f	http://www.neb.com/nebecomm/tech_reference/polymerases/properties_dna_polymerases.asp#.UBhsg7TbByo

Name	Resistance	Description
pZDonor 1-GTW-2	Amp	Position vector for position 1 - 2
pZDonor 2-GTW-3	Amp	Position vector for position 2 - 3
pZDonor 3-GTW-4	Amp	Position vector for position 3 - 4
pZDonor 4-GTW-5	Amp	Position vector for position 4 - 5
pZDonor 5-GTW-6	Amp	Position vector for position 5 - 6
pZDonor 6-GTW-7	Amp	Position vector for position 6 - 7
pZDonor 7-GTW-8	Amp	Position vector for position 7 - 8
pZDonor 9-GTW-10	Amp	Position vector for position 8 - 9
pZDonor 11-GTW-12	Amp	Position vector for position 11 - 12
pKan_Seq2-cHS4-Seq2-ISceI-HAR-Kan-SeqX	Kan	Adapter vector for terminating a gene circuit with UNS 2
pKan_Seq3-cHS4-Seq2-ISceI-HAR-Kan-SeqX	Kan	Adapter vector for terminating a gene circuit with UNS 3
pKan_Seq4-cHS4-Seq2-ISceI-HAR-Kan-SeqX	Kan	Adapter vector for terminating a gene circuit with UNS 4
pKan_Seq5-cHS4-Seq2-ISceI-HAR-Kan-SeqX	Kan	Adapter vector for terminating a gene circuit with UNS 5
pKan_Seq6-cHS4-Seq2-ISceI-HAR-Kan-SeqX	Kan	Adapter vector for terminating a gene circuit with UNS 6
pKan_Seq7-cHS4-Seq2-ISceI-HAR-Kan-SeqX	Kan	Adapter vector for terminating a gene circuit with UNS 7
pKan_Seq8-cHS4-Seq2-ISceI-HAR-Kan-SeqX	Kan	Adapter vector for terminating a gene circuit with UNS 8
pKan_Seq9-cHS4-Seq2-ISceI-HAR-Kan-SeqX	Kan	Adapter vector for terminating a gene circuit with UNS 9
pKan_Seq10-cHS4-Seq2-ISceI-HAR-Kan-SeqX	Kan	Adapter vector for terminating a gene circuit with UNS 10
pKan_Seq11-cHS4-Seq2-ISceI-HAR-Kan-SeqX	Kan	Adapter vector for terminating a gene circuit with UNS 11
pKan_Seq12-cHS4-Seq2-ISceI-HAR-Kan-SeqX	Kan	Adapter vector for terminating a gene circuit with UNS 12
pJazz-Swing	Cm	Linear carrier vector
pBAC_1-X	Cm	Circular BAC-based carrier vector
pENTR_L4_TRE-tight_R1	Kan	pENTR containing a rtTA/Doxycycline-inducible promoter
pENTR_L4_hEF1a_R1	Kan	pENTR containing the human EF1a promoter (constitutive expression)
pENTR_L4_CAG_R1	Kan	pENTR containing the CAG promoter

		(constitutive expression)
pENTR_L1_RheoAct-2A-RheoRec_L2	Kan	pENTR encoding the two Rheo receptors
pENTR_L1_EYFP_L2	Kan	pENTR encoding EYFP
pENTR_L1_NLS-mKate_L2	Kan	pENTR encoding a nuclear-localizing mKate (red fluorescent protein)
pENTR_L1_H2B-ECFP_L2	Kan	pENTR encoding a nuclear-localizing ECFP
pENTR_L1_Bla_L2	Kan	pENTR encoding the Blasticidin resistance gene
pENTR_L1_mmKate_L2	Kan	pENTR encoding a membrane-localizing mKate (red fluorescent protein)

Supplementary Table 2. Components (plasmids) used to construct gene circuits. Amp: Ampicillin; Kan: Kanamycin; Cm: Chloramphenicol.

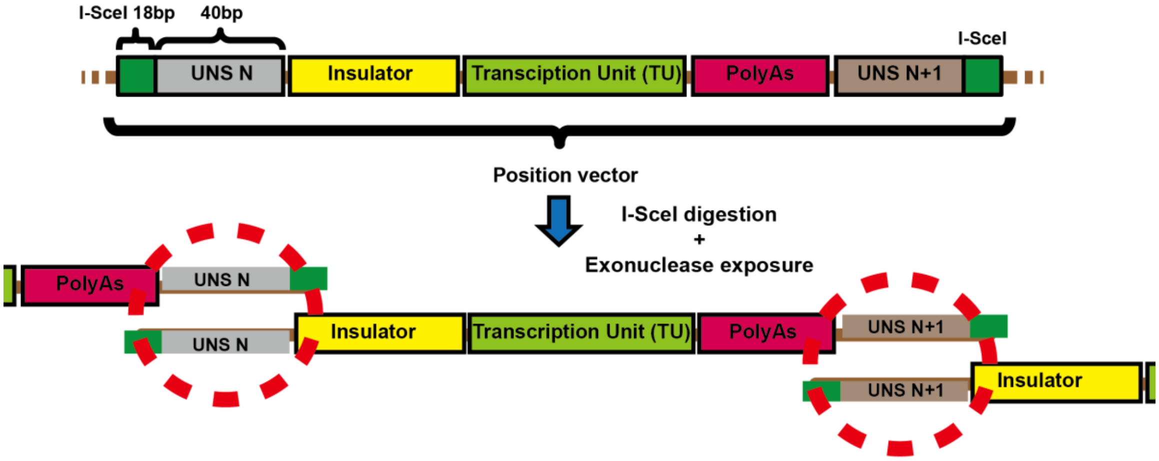

(a)

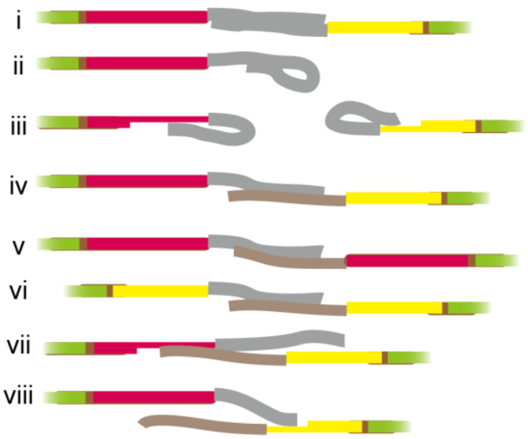

(b)

Supplementary Figure 1. Design of position vector and the unique oligonucleotide sequence (UNS). **(a)** Schematic design of a basic assembly unit. Exposure, annealing and repair of cognate UNSs during assembly reaction occurs within the dashed circles. **(b)** The following annealing reactions are taken into account in designing UNSs. From top to bottom: (i) pairing between cognate UNSs, (ii) duplex formation within UNS or (iii) between UNS and sequence in close proximity exposed during assembly reaction, (iv) pairing between non-cognate UNSs, (v, vi) pairing between non-cognate sense-strand UNSs, (vii, viii) pairing between UNS and sequence in close proximity exposed during assembly reaction.

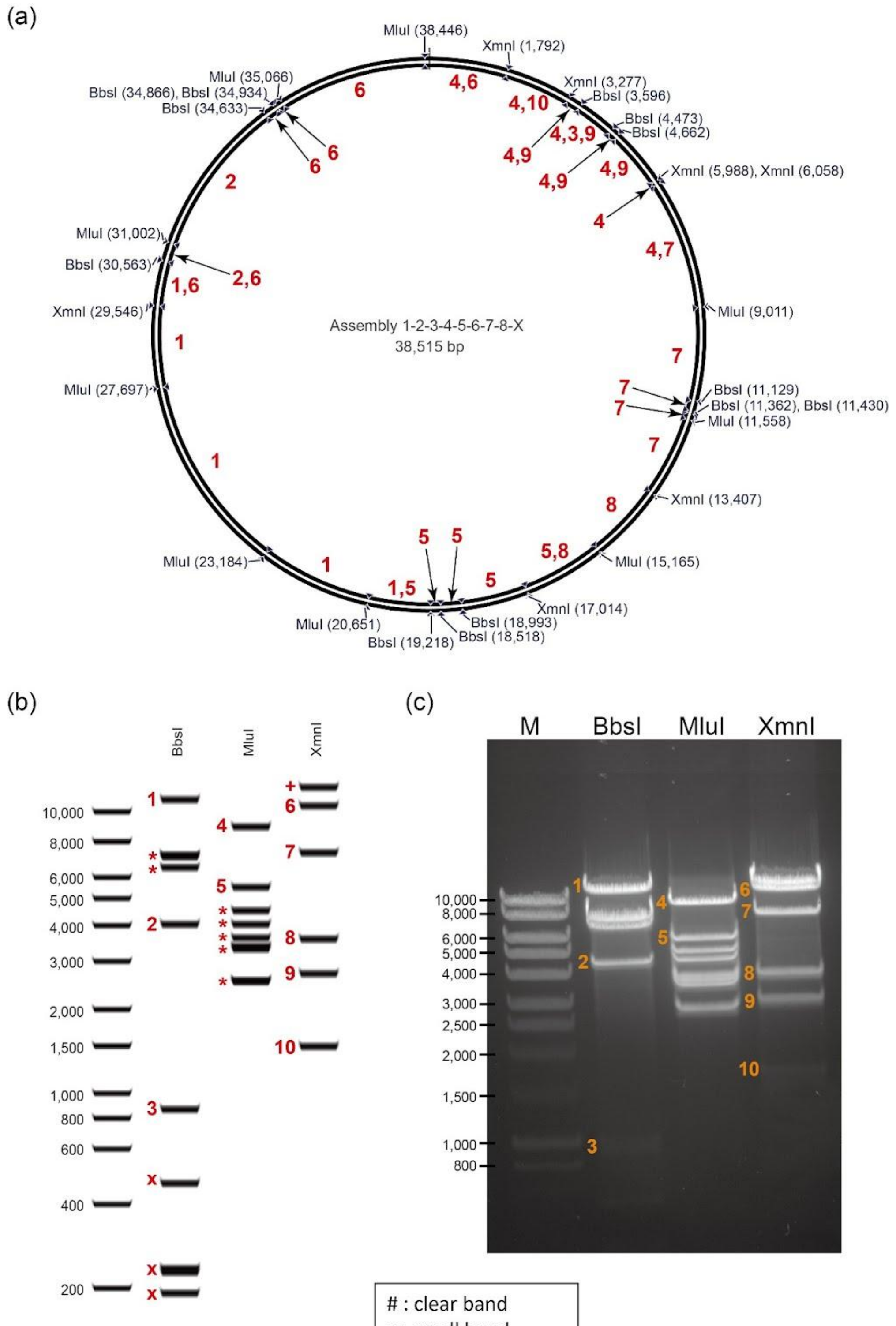
(a)
MluI (38,446)
XmnI (1,792)
MluI (35,066)
BbsI (34,866), BbsI (34,934)
BbsI (34,633)
XmnI (3,277)
BbsI (3,596)
BbsI (4,473)
BbsI (4,662)
XmnI (5,988), XmnI (6,058)
MluI (31,002)
BbsI (30,563)
XmnI (29,546)
MluI (9,011)
Assembly 1-2-3-4-5-6-7-8-X
38,515 bp
MluI (27,697)
BbsI (11,129)
BbsI (11,362), BbsI (11,430)
MluI (11,558)
XmnI (13,407)
MluI (23,184)
MluI (15,165)
XmnI (17,014)
MluI (20,651)
BbsI (18,993)
BbsI (19,218)
BbsI (18,518)
(b)
BbsI
MluI
XmnI
10,000
8,000
6,000
5,000
4,000
3,000
2,000
1,500
1,000
800
600
400
200
100
(c)
M
BbsI
MluI
XmnI
10,000
8,000
6,000
5,000
4,000
3,000
2,500
2,000
1,500
1,000
800
# : clear band
x : small band
+ : large band
* : overlapping band

Supplementary Figure 2. A restriction enzyme search algorithm for verifying a large plasmid with high resolution. The restriction pattern by an enzyme may contain large bands, small bands, overlapping bands and clear bands. Large bands are those that run slower than the highest band of a DNA ladder. Small bands are those that are too small in mass to be seen in a gel image. Overlapping bands are those that run too close to another band, in which case it is difficult to distinguish one or another under normal gel running and exposure conditions. Clear bands are bands that can be clearly identified in a gel image. (a) An example showing restriction mapping. A plasmid is computationally fragmented by cut sites of selected restriction enzymes BbsI, MluI, and XmnI. Each fragment is mapped to one or more clear bands (indicated by numbers, in consistency with b) in restriction patterns by the selected restriction enzymes. (b) Bands from restriction patterns of the plasmid shown in a are labeled with, number: clear bands, *: overlapping bands, #: big bands, x: small bands. The restriction pattern is generated using Geneious Pro(20) (c) Actual restriction pattern of the same plasmid shown in a. Only clear bands are labeled with numbers in consistency with b.

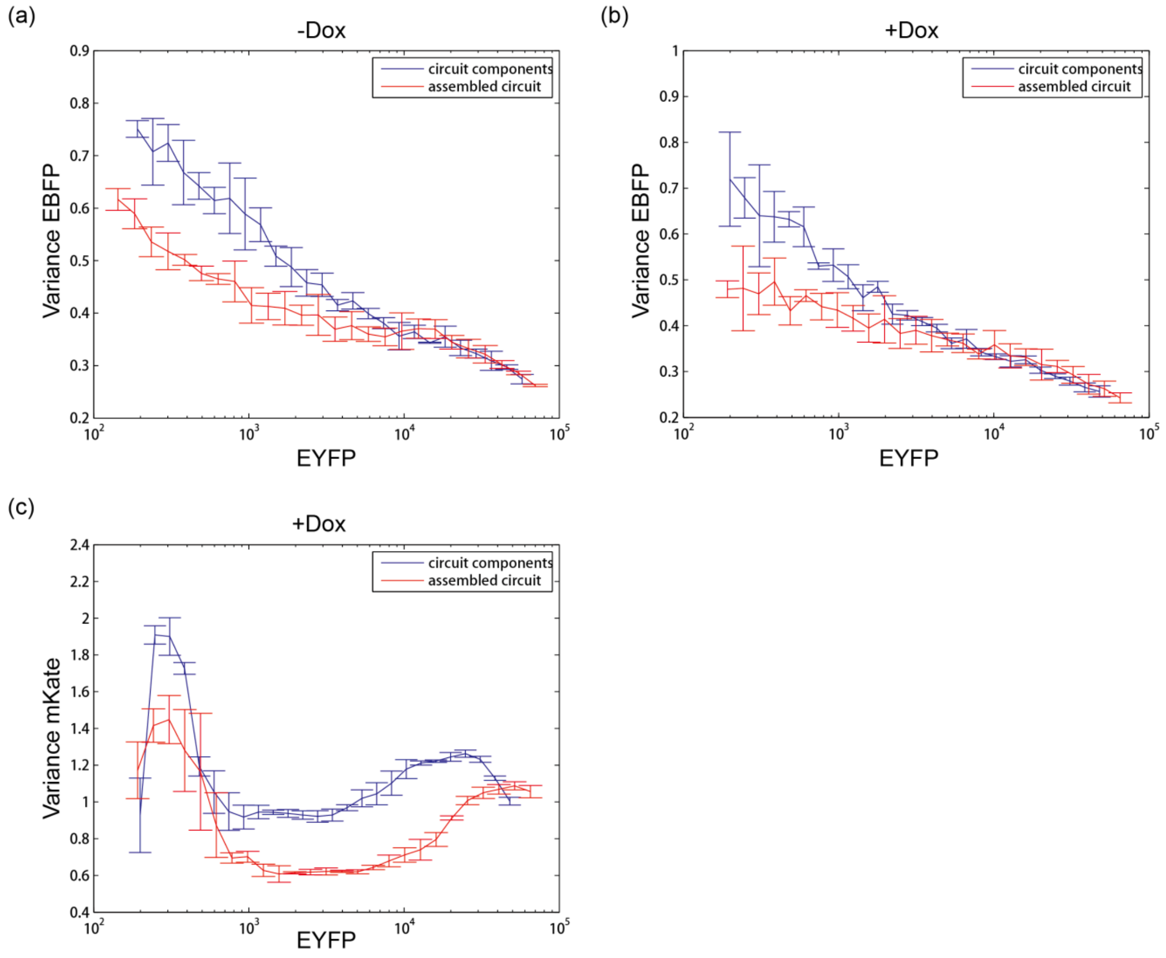

Supplementary Figure 3. The transfected vector of an assembled circuit exhibits less noise than the co-tranfected plasmids containing the same circuit components. Circuit components
(co-transfection) or assembled circuit (single vector transfection) described in Figure 2b were transiently transfected into HEK293FT cells. Samples were analyzed in flow cytometry 48 hours post transfection (+/- Doxycycline induction). Fluorescence is measured in arbitrary units. The kernel density estimation technique(21) was employed to find 2D density distribution in two-color (EBFP-EYFP, and mKate-EYFP) plots using flow cytometry data. Once the 2D probability was obtained, mean and variance of EBFP and mKate for each EYFP level were calculated and plotted. (a) EBFP-EYFP plot for samples without Doxycycline. (b) EBFP-EYFP plot for samples with Dox. (c) mKate-EYFP plot for samples with Doxycycline. The trend is the same when using EBFP as the transfection marker (data not shown) and performing the same analysis.

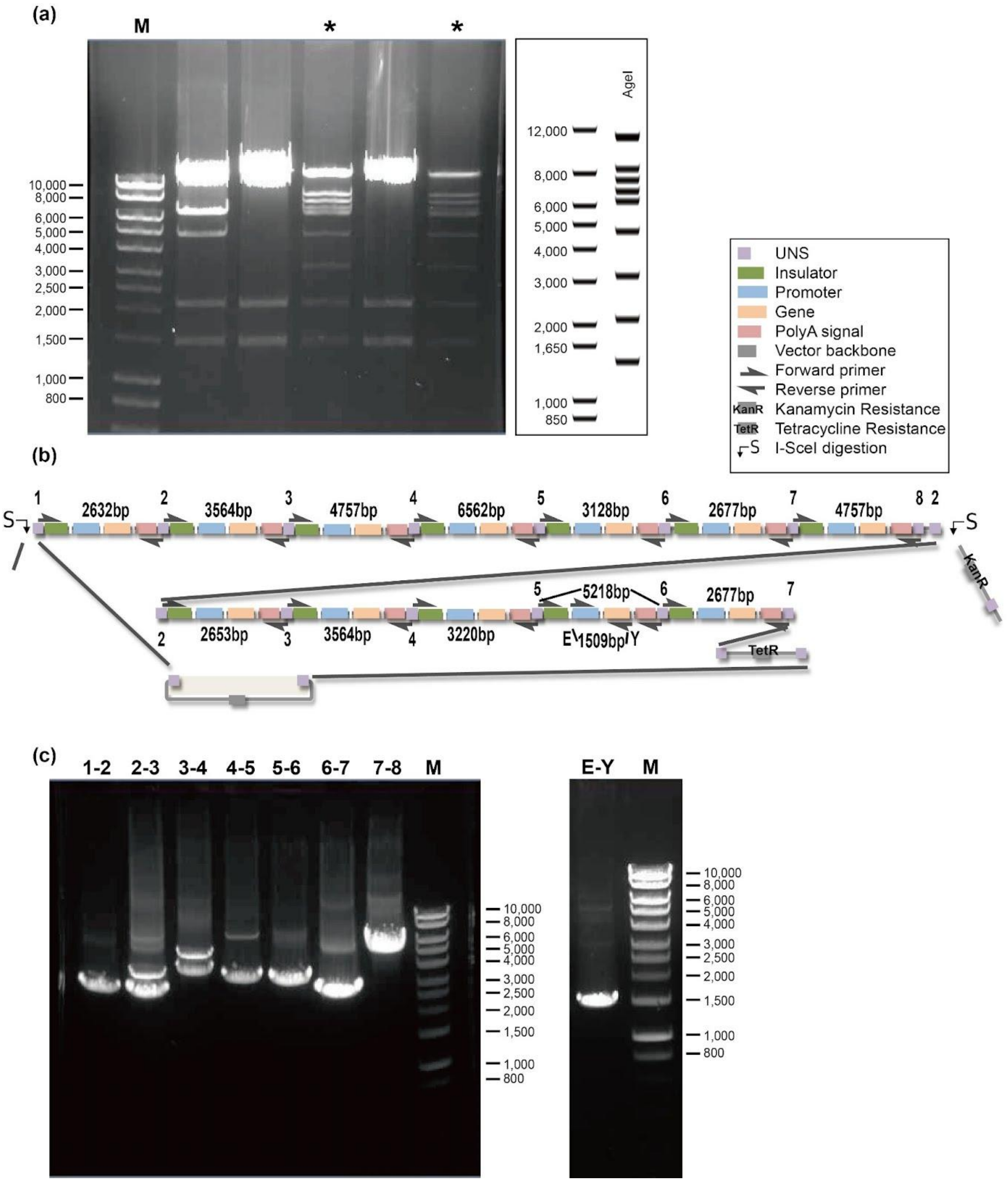

Supplementary Figure 4. Hierarchical assembly of a 12 transcription units (TU) gene circuit (62.7kbp) combines a 7 TU circuit (assembled in the first round) with additional 5 TUs in the second round assembly. **(a)** Left: gel electrophoresis image of a restriction digestion of a 12 TU circuit. *: correct restriction pattern. Right: expected AgeI digestion pattern *in-silico*. **(b)** Circuit composition, primer binding sites and expected PCR products size for the 12 TU circuit. The first line represents TUs assembled in the first round. It is released from its vector backbone and Kanamycin resistance adapter by I-SceI digestion. The second line represents hierarchically added TUs, followed by a Tetracycline resistance adaptor and a carrier vector. purple rectangle: UNS, green rectangle: insulator, blue rectangle: promoter, orange rectangle: CDS, pink rectangle:

polyadenylation signal. Black arrows above rectangles indicate forward primers. Black arrow below rectangles indicate reverse primers. Expected PCR product size for a pair of primers is written between primers. Numbers above UNS indicate its sequence. E: EYFP, Y: Hygromycin resistance gene. Primer pairs 2-3, 3-4, 4-5, 5-6, 6-7 amplify TUs assembled from both the first round and the second round in multiplex PCRs. **(c)** Left: PCR products amplified using primer pairs described in b. Note that PCR product for the second 5-6 (5218bp) is merely visible in the gel image. It is likely that amplification is highly biased toward the first 5-6 (3128bp). Right: To verify that the second 5-6 is present, its template EYFP-2A-Hygro is amplified.

Supplementary methods.

Algorithm and parameters for designing the unique oligonucleotide sequences.

```
procedure Stochastic Local Search for UNS Design
   input: Number of UNS, length of UNS,
          search pool size, set of constraints (C),
          crossover rate β,
          mutation rate λ
   output: Set of UNS that satisfy C
   S := ∅
   P := initial random pool of candidate UNSs
   for l := 1 to maxIterations do
      for i := 1 to maxTries do
         for j := 1 to size(P) do
            if P(j) satifsfies all constraints then
               add P(j) to S
               if S contains enough UNSs then
                  return S
               end if
            end if
            for k := 1 to size(S) do
               AF(k) is increased by the number of alignments fails for UNS(k) ∈ S in current iteration
            end for
            θ := inverse proportional to the # of constraints that candidate ∈ P violate
            for k := 1 to size(P) do
               select cU1, cU2 ∈ P with probability θ respectively
               cÛ := new candidates from crossover(cU1, cU2, β)
               add P̂ new candidates from cÛ by mutating bases with rate λ
            end for
            if P̂ has indentical candidates within P ∪ P̂ then
               remove identical candidates from P̂
               add random candidate to fill P̂
            end if
            P := P̂
         end for
      end for
      remove UNS ∈ S that has maximum AF for all UNSs ∈ S
   end for
   return S
end
```

Parameter Constraints	Value
Length of UNS	40
Length of UNS head region tested in alignment	7
Smith-Waterman alignment threshold for whole sequence	50
Smith-Waterman alignment threshold for sequence head region	25
Restriction sites	AgeI, XbaI
Secondary structure dG	-5 kcal/mol
Annealing temperature	65C - 75C
GC content percentage	40% - 80%
Crossover rate	0.9
Mutation rate	0.1
Random sequence pool size	10

UNSs guide the position-vector digested fragments assembly into a full circuit. Nonspecific annealing (Figure S1) can occur between non-complementary UNSs, UNS and the reverse complementary (rc) UNSs, UNSs and insulator (exposed by the exonuclease), UNSs and Polyadenylation Signal (exposed by the exonuclease).

Therefore, it was necessary to design UNSs such that the probability of any nonspecific annealing other than between complementary UNSs is low enough for an assembly of many position vectors simultaneously. Computationally, the Smith–Waterman local alignment score was used as a measure for the annealing probability. The probability of unspecific annealing is proportional to the Smith–Waterman local alignment score of the involved sequences - the higher the alignment score, the higher the probability that two sequences will anneal.

Because the free leading ends of UNSs can serve as the seeding region for unspecific annealing, even though the entire sequences may not have high alignment scores, alignment constraints were applied to the 7 bp leading head on both sides of UNSs

In addition, to avoid nonspecific annealing, several other constraints need to be met: Firstly, it is necessary to ensure that all complementary UNSs anneal easily within a certain temperature range. This condition was determined by the annealing temperature of the UNSs. Secondly, the secondary structure within the UNS has to be minimized. The secondary structure of the UNSs can affect the efficiency of annealing between two matching sequences and, hence, the efficiency of assembly. The GC base pair content of an UNS was set to be within a specific range. Thirdly, the UNS needs to be free of some restriction sites so as to allow easy modification to the assembly vectors by restriction cloning. The box lists the constraints used in the design of the UNS and the parameters used for the UNS searching.

We designed a stochastic local search algorithm for an iterative UNSs discovery and optimization. In each iteration, the algorithm starts with a pool of random sequences. Each sequence from the pool is aligned to the UNSs already found. The best sequence, with alignment scores lower than a threshold is screened for its secondary structure, melting temperature, and restriction sites. If it meets all constraints, it is added as a new UNS to the pool of existing UNSs. The algorithm repeats the process until a desired number of UNSs are found. If no sequence from the random pool meets all requirements, a few sequences with low alignment scores are picked. A new search pool is then generated by recombining and mutating those selected sequences, and by adding newly generated random sequences, in a process similar to a genetic algorithm. Subsequently, the search procedure iterates. If the iteration number exceeds a predefined maximum, it is assumed that some of previously found UNSs are too restrictive for addition of a new sequence. In this case, the UNS that failed in most alignment-test failures is removed, and a new search process started. 13 new UNS were designed with this algorithm. The quality of the design sequence is measured by the ΔG gap, the smallest energy gap between any pair of matching UNSs and mis-matching UNSs(22–24). The ΔG gap for the designed sequence is -32 kcal/mol.

Algorithm for automated enzyme selection for restriction mapping

```
procedure Exhaustive restriction enzyme search for optimal restriction mapping
    input: Vector sequence S
           Enzyme list
           Enzyme methylation sensitivity
           Standard DNA ladder L
    output: A list of restriction enzymes that ranked by restriction mapping coverage
    E := initial enzyme list
    E := E + all possible double digestion enzymes
    for i := 1 to size(E) do
        C(i) := find all cut positions by E(i)
        if E(i) is DAM or DCM sensitive then
            remove DAM, DCM cut positions from C(i)
        end if
        F(i) := length of restriction fragments calculated from C(i)
        B(i) := band positions on gel, transformed F(i) according to L
        label each band b ∈ B(i)
            b is labeled as BAND_LARGE, if b > max(L)
            b is labeled as BAND_SMALL, if m.w.(b) < m.w.(min(L)) //m.w. : molecular weight
            b is labeled as BAND_OVERLAP, if ∃ b' such that |b-b'| < minimal visible separation of L
            b is labeled as BAND_CLEAR otherwise
    end for
    T := possible combinatorials of E for separate digestions
    for i := 1 to size(T) do
        for j := 1 to size(E used in T) do
              map S to B(i)
        end for
        score(i) := total length of intervals ∈ S that cannot be mapped to any b labeled as BAND_CLEAR
    end for
  rank T according to score
  return T
end
```

Assembled plasmids are usually larger than 10 kilobases. The restriction pattern of a single enzyme digest usually produces multiple bands, where one or more belong to the following categories: unclear bands (large bands, small bands, overlapping bands) and clear (usable) bands. Large bands are those that run slower than the highest band of a DNA ladder. Small bands are those that are too small in mass to be seen in a gel image. Overlapping bands are those that run close to another band, so that it is difficult to distinguish one or another in a normal gel running and exposure conditions. Clear bands are bands that can be clearly identified in a gel image. Most laboratories use general-purpose DNA ladders covering ~ 200 - 10’000 base pairs, for example the Bioline

Hyperladder I shown in Supplemental Figure S2b With this type of ladder, we estimate the ascertainable band to be less 10 kbp in size and to be more than 20 ng in mass.

A restriction map of a large construct by a single enzyme often contains unclear bands, as defined above. However, it is in most cases possible to map a construct fully with multiple enzymes in the same or in separate digests. Construct fragments mapped to unclear bands in a restriction pattern by one enzyme may be mapped to clear bands in restriction pattern by another enzyme. In order to map such large constructs with a 1 kbp ~ 2 kbp resolution, we designed an algorithm to automate the enzyme selection.

The algorithm takes in construct base-pair sequence and the list of restriction enzymes accessible to the laboratory as an input. The restriction sites for each enzyme are found by regular expression matching. Double digestions are performed by taking the union of the cut sites by two individual enzymes. If the bacterial cloning strain used for propagating the construct contains Dam or Dcm methylases, methylated sites are removed. Next, the relationship between band-size and band-position in an actual gel image is estimated by running a ladder of choice and analyzing its gel image. The *in silico* restriction pattern for each enzyme is estimated by interpolation based on the band length - position relationship of the ladder. Here, we use Hyperladder I as DNA ladder. For restriction analysis in which 1.5ug sample is digested per enzyme and run on 1% agarose gel for 30 min, criteria for clear bands are: less than 10 kbp, more than 20 ng in mass, and a separation of 10.41 *in silico*. Every band for all enzymes is labeled as large band, small band, overlapping band, or clear band based on the previously described criteria.

The algorithm then evaluates all possible two/three enzyme combinations. For each combination, the construct sequence is fragmented at all cut sites. An example is shown in Supplementary Figure S2, where a plasmid is cut with BbsI, MluI and XmnI. The program iterates through all fragments and finds (mapping) which ‘clear’ band covers a specific fragment. The search results are ranked by the total number of fragments unmapped or ranked by the summed length of unmapped fragments.

Plasmids

pLV_CAG_CN-2A-CN: The cDNA encoding the two zinc finger nucleases(25) for the AAVS1 locus separated by a 2A tag was synthesized (GeneArt, Regensburg, Germany) and PCR-amplified with attB1/attB2 tags using the primers oPG608b/oPG609b. Upon gel-extraction, the PCR-product was recombined into a pENTR_L1_L2 vector using BP clonase (Life Technologies, Carlsbad CA), yielding the pENTR_L1_CN-2A-CN_L2 vector. In a next step, pENTR_L1_CN-2A-CN_L2 and pENTR_L1_CAG_L2 were recombined into pLV_R4R2_GTW using the LR clonase II plus (Life Technologies, Carlsbad CA), resulting in pLV_CAG_CN-2A-CN. **pZDonor_Seq1-FseI-SeqX:** XbaI-Seq1-FseI-SeqX-MfeI was annealed from oligos o-YQ303, o-YQ304 which gave XbaI and MfeI compatible overhangs. XbaI-Seq1-FseI-SeqX-MfeI was inserted into pGTW4c cut with XbaI/MfeI. **pZDonor-L_ISceI-Seq1-FseI-SeqX:** MfeI-PciI was annealed from oligos o-YQ353, o-YQ354, which gave MfeI and PciI compatible overhangs. It was then inserted into pZDonor_ISceI-Seq1-FseI-SeqX cut with MfeI/PciI. **pZDonor_ISceI-Seq1-FseI-SeqX:** XbaI-ISceI-Seq1-FseI was annealed from oligos o-YQ331, o-YQ332 which gave XbaI and FseI compatible overhangs. XbaI-ISceI-Seq1-FseI was inserted into pZDonor_Seq1-FseI-SeqX cut with XbaI/FseI. **pZDonor_Seq1-PacI-SeqX:** XbaI-Seq1-PacI-SeqX-MfeI was annealed from oligos o-YQ308, o-YQ309 which gave XbaI and MfeI compatible overhangs. XbaI-Seq1-PacI-SeqX-MfeI was inserted into pGTW4c cut with XbaI/MfeI. **pZDonor I-SceI L:** HindIII-ISceI-XhoI was annealed from oligos o-YQ205, o-YQ206, which gave HindIII and XhoI compatible overhangs. It was then inserted into pGTW4c cut with XbaI/MfeI. **pZDonor I-SceI R:** AgeI-ISceI-XhoI was annealed from oligos o-YQ207, o-YQ208, which gave AgeI and XhoI compatible overhangs. It was then inserted into pZDonor I-SceI L cut with AgeI/XhoI. **pZDonor Position Vectors:** cHS4-Gateway selection cassette-rabbit polyadenylation signal was amplified from pGTW4c with oligos listed in the table below. It was a nested PCR. The first step used the first set of oligos and the product was used as the template for the second step PCR with the second set of oligos. The PCR products were digested with AgeI/PacI and ligated into pZDonor I-SceI R cut with AgeI/PacI. The vectors were selected Cm. The vectors were diluted into 10 fmol for Gateway reaction. **pLV_R4R2_GTW:** The R4_GTW_R2 cassette (1743 bp) was PCR-amplified with oPG240/oPG241, cut with PacI/MfeI and cloned into PacI/EcoRI-cut pFHGUBW(26). **pKan_cHS4-HAR-Kan-SeqX:** The Kanamycin resistance gene was amplified from pDONR221P1P2 vector (Life Technologies, Carlsbad CA) with oligos o-YQ313, o-YQ314. The pUC origin of replication was amplified with o-YQ321, o-YQ322 from the same vector. They were digested with XbaI/XhoI and ligated. cHS4-HAR cassette was digested from pGTW4c with XbaI/SacII and inserted into the ligated vector cut with XbaI/SacII. **pKan_cHS4-Seq2-ISceI-HAR-Kan-SeqX:** SalI-Seq2-ISceI-EagI was annealed from oligos o-YQ333, o-YQ334, which gave SalI and EagI compatible overhangs. It was then inserted into pKan_cHS4-HAR-Kan-SeqX cut with SalI/EagI. **Adaptor Vectors:** The adaptor vectors were constructed by annealing oligos listed in the table below. The annealed oligos had XbaI and MfeI compatible overhangs. They were inserted into pKan_cHS4-Seq2-ISceI-HAR-Kan-SeqX cut with XbaI/MfeI. **pGTW4b:** The Gateway selection cassette was amplified from pLenti6 (Life technologies, Carlsbad CA) using the oligos oPG452/453, cut with XhoI and MluI and inserted into XhoI/MluI-cut pAAVS1-CAGGS-eGFP(25), resulting in GTW4b. **pGTW4c:** The tandem repeated cHS4 core insulators were extracted from pNI-CD (courtesy of G. Felsenfeld, NIH, MD) using EcoRI and KpnI and ligated into MfeI/KpnI-cut GTW4b. **pHL_SA-2A-Puro_1-X_HR:** The splice acceptor, puromycin gene and polyadenylation sequences were amplified from pAAVS1-SA-2A-Puro[2] using oPG880/oPG881, digested with NheI and ligated into XbaI-

cut pGTW4c, creating pHL_SA-2A-Puro_1-X_HR. **pHL_SA-2A-Bla_1-X_HR:** The bleomycin resistance gene was amplified with a splice acceptor and a 2A tag from pLenti6 using oPG950/oPG953 and then oPG952/oPG953, digested with BglII and XbaI and ligated into BglII/XbaI-cut pHL_SA-2A-Puro_1-X_HR. **pJazz-PreBop, pJazz-BeBop:** Subsequently we amplified the HL_SA-2A-Puro_1-X_HR or the HL_SA-2A-Bla_1-X_HR cassette from pHL_SA-2A-Puro_1-X_HR respectively pHL_SA-2A-Bla_1-X_HR using oPG450f/oPG451, digested it with NotI and cloned it into the NotI-cut pJazz vector (Lucigen, WI), yielding pJazz-PreBop (Puromycin resistance) and pJazz-BeBop (Basticidin resistance). **pJazz-Swing:** A 304 bp cassette was digested with NheI and FseI from pZDonor-L_ISceI-Seq1-FseI-SeqX and ligated into the NheI/FseI cut pJazz-PreBop, yielding pJazz-Swing. **pBAC_BP_1-X:** NotI-cut pSMART-BAC (Lucigen, Middleton WI 53562, USA) was ligated with the two annealed oligos pZX_1 and pZX_2, inserting a multiple cloning site containing an AscI site into the vector and creating pZ295. The 1-X_2xcHS4 sequence (754 bp) was cut out of pZDonor_Seq1-PacI-SeqX using XbaI/NotI and ligated into NheI/NotI-cut pZ295, creating pBAC_1-X. pBAC_1-X was then PCR-amplified using oPG1592/1593 resulting in a fragment 8430 bp in size, which was fused to a PCR-amplified Puromycin gene with it's polyadenylation sequence (PCR with oPG1590/1591, 1430 bp in size) by applying the Gibson reaction mix, creating pBAC_BP_1-X. pENTR_L4_TRE-tight_R1 was constructed by PCR-amplifying TRE-tight from pTRIPZ (Openbiosystems, Lafayette, Co). **pENTR_L4_hEF1a_R1** was constructed by PCR-amplifying hEF1a from pLV-hEF1a-IRES2- Puro. **pENTR_L1_rtTA3_L2** was constructed by PCR-amplifying rtTA3 from pTRIPZ. **pENTR_L1_acyl-mKate2_L2** was constructed by adding the palmitoylation signal from mEYFP in pBrainbow-1.1M(27) to the N-terminus of mKate2 (Evrogen, Moscow, Russia) **pENTR_L4_CAG_R1** was constructed by extracting the CAG promoter using PacI/EcoRI from pCAG-DsRed-FF5 and cloning it PacI/EcoRI-cut pENTR_L4_MCS_R1. **pENTR_L1_RheoAct-2A-RheoRec_L2** was constructed by PCR-ampflifying RheoAct and RheoRect from pNEBR-R1 (NEB, Ipswich MA). **pENTR_L1_EYFP_L2** was by amplifying EYFP from pBrainbow-1.1M. **pENTR_L1_H2B-ECFP_L2** was constructed by cloning the sequence encoding the human histone H2B to the reading frame encoding a cyan fluorescent protein. **pENTR_L1_Bla_L2** was constructed by PCR-amplifying and subcloning the *bla* resistance gene. **pENTR_L1_NLS-mKate_L2** was constructed by adding the SV40 NLS ("PPKKKRKV") to the N-terminus of mKate using PCR-amplification. **pENTR_L1_mmKate_L2** was constructed by adding an N-terminal palmitoylation signal to the sequence encoding and mKate2 (Evrogen, Moscow, Russia).

Cell culture

HEK293FT (Invitrogen, Carlsbad CA) cells were cultured in supplemented DMEM according to its manual. Chemical DNA transfection was performed using Qiagen SuperFect Transfection Reagent (QIAGEN, Hilden, Germany) or Metafectene Pro (Biontex, Martinsried, Germany). In brief, 800,000 cells were seeded into a 10 cm2 well and immediately transfected with a total of 2 µg DNA. The medium was replaced six hours post-transfection. To induce the Tet-On system, Doxycycline (Clontech, Mountain View, CA) was added to culture media at 1 µg/mL. To induce the Rheo system, RHL/Genostat (EMD Millipore, Burlington, MA) was added to the cell culture at a final concentration of 5 nM. Targeted integration into the AAVS1 locus: The carrier vector was stably integrated(28) into the chromosome of HEK293 cells and stable clones have been selected with 1ug/ml of Puromycin (Manuscript in preparation).

Microscope imaging

Images were taken using a Leica TCS SP5 II 405UV confocal microscope (Leica Microsystems, Bannockburn, IL). Images were acquired using a sequential scan. 1st scan: Excitation/Laser lines: 488 nm, Emission: 495-539 nm; 2nd scan: Excitation/Laser lines: 458 and 543 nm, Emission: 462-487 and 547-800 nm respectively.

Flow cytometry measurement

Flow cytometry measurement was carried out on BD LSR II in Koch Institute Flow Cytometry Core at MIT. Data was collected in BD FACSDiva software and analyzed in Flowjo (Tree Star, Inc. Ashland, OR)

References

20. Drummond AJ, Ashton B, Buxton S, Cheung M, Cooper A, Duran C, Field M, Heled J, Kearse M, Markowitz S, Moir R, Stones-Havas S, Sturrock S, Thierer T,W.A. (2011) Geneious v5.4. *Available from http://www.geneious.com*.

21. Botev,Z.I., Grotowski,J.F. and Kroese,D.P. (2010) Kernel density estimation via diffusion. *The Annals of Statistics*, **38**, 2916–2957.

22. Shortreed,M.R., Chang,S.B., Hong,D., Phillips,M., Campion,B., Tulpan,D.C., Andronescu,M., Condon,A., Hoos,H.H. and Smith,L.M. (2005) A thermodynamic approach to designing structure-free combinatorial DNA word sets. *Nucleic acids research*, **33**, 4965–77.

23. Andronescu,M., Zhang,Z.C. and Condon,A. (2005) Secondary structure prediction of interacting RNA molecules. *Journal of molecular biology*, **345**, 987–1001.

24. Andronescu,M. (2003) RNAsoft: a suite of RNA secondary structure prediction and design software tools. *Nucleic Acids Research*, **31**, 3416–3422.

25. Hockemeyer,D., Soldner,F., Beard,C., Gao,Q., Mitalipova,M., DeKelver,R.C., Katibah,G.E., Amora,R., Boydston,E.A., Zeitler,B., et al. (2009) Efficient targeting of expressed and silent genes in human ESCs and iPSCs using zinc-finger nucleases. *Nature biotechnology*, **27**, 851–7.

26. Xie,Z., Wroblewska,L., Prochazka,L., Weiss,R. and Benenson,Y. (2011) Multi-Input RNAi-Based Logic Circuit for Identification of Specific Cancer Cells. *Science*, **333**, 1307–1311.

27. Livet,J., Weissman,T.A., Kang,H., Draft,R.W., Lu,J., Bennis,R.A., Sanes,J.R. and Lichtman,J.W. (2007) Transgenic strategies for combinatorial expression of fluorescent proteins in the nervous system. *Nature*, **450**, 56–62.

28. Brown,W.R.A., Lee,N.C.O., Xu,Z. and Smith,M.C.M. (2011) Serine recombinases as tools for genome engineering. *Methods (San Diego, Calif.)*, **53**, 372–9.
