# Supplementary material for: Rapid, modular and reliable construction of complex mammalian gene circuits
Source: Nucleic Acids Res. 2013 Jul 11;41(16):e156. doi: 10.1093/nar/gkt605 (PMC3763561; doi:10.1093/nar/gkt605)
Supplement: Supplementary Data [file supp_gkt605_nar-01230-met-k-2013-File005.pdf]

oPG240	GAATTCTTAATTAACAACTTTGTATAGAAAAGTTGAACGAG
oPG241	GAATCCCAATTGCCGGAATTCACCACTTTGTACAAGAAAGCTG
oPG450f	GGGAATTCGCGGCCGCGGCCATTACGGCCTGCTTTCTCTGACCAGCATTCTCTCC
oPG451f	GGGTTCCTGGCTCTGCTCTCCACGGCCGCCTCGGCCGCGGCCGCGAATTCCC
oPG452	CACCGCTCGAGACAATTGCATCGATGGTACCGTATCGATGTCGACGTTAACGCTAGTG
oPG453	CTCGTTCAGCTTTCTTGTACAAAGTGGTACGCGTGAATTCCCATG
oPG608b	GGGGACAAGTTTGTACAAAAAAGCAGGCTGACTGCCACCATGGCCCCCAAGAAGAAGCGG
oPG609b	GAAATCAATTTCTGAGGCGCGCCTACCCAGCTTTCTTGTACAAAGTGGTCCCC
oPG880	GGGAATTCCTAGCTAGCAAGCTTCTGACCTCTTCTCTTCCTCC
oPG881	GCTCTATGGGTCGACAGTACTAAGCTTGCTAGCTAGGGAATTCCC
oPG950	GTGACGTGGAGGAGAATCCCGGCCCTAGGCTCGAGATGGCCAAGCCTTTGTCTCAAGAAG
oPG952	GGGAATTCAGATCTGGCAGCGGAGAGGGCAGAGGAAGTCTTCTAACATGCGGTGACGTGGAGGAGAATCC
oPG1590	GGGCTTGTCGACGACGGCG
oPG1591	GGCACAGTCAATGAAGTTTGAAAACCAATAAG
oPG1592	GGTTTACCGAGCTCTTATTGGTTTTCAAACTTC
oPG1593	CCTGACGACGGAGACCGCCGTCGTCGACAAGCCCCAAGGCCGCTTCTATAGTGTCACCTA
o-YQ205	AGCTTATTACCCTGTTATCCCTAACCGGTCAGGACACCAC
o-YQ206	TCGAGTGGTGTCCTGACCGGTTAGGGATAACAGGGTAATA
o-YQ207	CCGGTCAGGACACCATTAATTAATAGGGATAACAGGGTAATC
o-YQ208	TCGAGATTACCCTGTTATCCCTATTAATTAATGGTGTCCTGA
o-YQ209	ATACTTGAACCGGTTTACCGAGCTCTTATTGGTTTTCAAACTTCATTGACTGTGCCAAGC
o-YQ210	GAGCTCTTATTGGTTTTCAAACTTCATTGACTGTGCCAAGCTTCTGACCTCTTCTCTTCC
o-YQ211	TAAGTAGATTAATTAAGGATCTTAAAAACATTATACAATACTACAAGCATAAAAACGCAC
o-YQ212	AACATTATACAATACTACAAGCATAAAAACGCACCAAGCTTAGTACTGTCGATCCGCTGG
o-YQ213	ATACTTGAACCGGTGCGTTTTTATGCTTGTAGTATTGTATAATGTTTTTAAGATCCAAGC
o-YQ214	TTTATGCTTGTAGTATTGTATAATGTTTTTAAGATCCAAGCTTCTGACCTCTTCTCTTCC
o-YQ215	TAAGTAGATTAATTAAGGCGTATAAAACATCTGGATAAGACGAGAGATTGGGTATTAGAC
o-YQ216	ACATCTGGATAAGACGAGAGATTGGGTATTAGACCAAGCTTAGTACTGTCGATCCGCTGG
o-YQ230	ATACTTGAACCGGTCTAATACCCAATCTCTCGTCTTATCCAGATGTTTTATACGCCAAGC
o-YQ231	ACCCAATCTCTCGTCTTATCCAGATGTTTTATACGCCAAGCTTCTGACCTCTTCTCTTCC
o-YQ232	ATACTTGAACCGGTGAATTCCCTTATGTGAGTGTAAAAGGCAGGCGAGTTTGTCCCAAGC
o-YQ233	CCCTTATGTGAGTGTAAAAGGCAGGCGAGTTTGTCCCAAGCTTCTGACCTCTTCTCTTCC
o-YQ234	ATACTTGAACCGGTTGCTTGCAAAAGCAGTAATTGGAAAGCACTCTCAAAGAATCCAAGC
o-YQ235	GCAAAAGCAGTAATTGGAAAGCACTCTCAAAGAATCCAAGCTTCTGACCTCTTCTCTTCC
o-YQ236	ATACTTGAACCGGTAGATAAGTTGATTTAGCCATAAAATATTGTTTCCGTGACCCCAAGC
o-YQ237	AGTTGATTTAGCCATAAAATATTGTTTCCGTGACCCCAAGCTTCTGACCTCTTCTCTTCC
o-YQ238	ATACTTGAACCGGTTCTGAGTCACGGCTTCATTGGCATTCCGTACAACGAACGTCCAAGC
o-YQ239	GTCACGGCTTCATTGGCATTCCGTACAACGAACGTCCAAGCTTCTGACCTCTTCTCTTCC
o-YQ240	TAAGTAGATTAATTAAGGGACAAACTCGCCTGCCTTTTACACTCACATAAGGGAATTCAC

o-YQ241	CGCCTGCCTTTTACACTCACATAAGGGAATTCACCAAGCTTAGTACTGTCGATCCGCTGG
o-YQ242	TAAGTAGATTAATTAAGGATTCTTTGAGAGTGCTTTCCAATTACTGCTTTTGCAAGCAAC
o-YQ243	AGAGTGCTTTCCAATTACTGCTTTTGCAAGCAACCAAGCTTAGTACTGTCGATCCGCTGG
o-YQ245	AAACAATATTTTATGGCTAAATCAACTTATCTACCAAGCTTAGTACTGTCGATCCGCTGG
o-YQ246	TAAGTAGATTAATTAAGGACGTTCGTTGTACGGAATGCCAATGAAGCCGTGACTCAGAAC
o-YQ247	TGTACGGAATGCCAATGAAGCCGTGACTCAGAACCAAGCTTAGTACTGTCGATCCGCTGG
o-YQ248	TAAGTAGATTAATTAAGGGGATGCATGGTGTTGTTTTACCGCTATAGGCTCTCTGAGGAC
o-YQ249	GGTGTTGTTTTACCGCTATAGGCTCTCTGAGGACCAAGCTTAGTACTGTCGATCCGCTGG
o-YQ303	CTAGAGGTTTACCGAGCTCTTATTGGTTTTCAAACTTCATTGACTGTGCCGGCCGGCCGGTATCACTAGTATTACAGAGGTAAGTTATAACAGTCGCCTAACCC
o-YQ304	AATTGGGTTAGGCGACTGTTATAACTTACCTCTGTAATACTAGTGATACCGGCCGGCCGGCACAGTCAATGAAGTTTGAAAACCAATAAGAGCTCGGTAAACCT
o-YQ308	CTAGAGGTTTACCGAGCTCTTATTGGTTTTCAAACTTCATTGACTGTGCCTTAATTAAGGTATCACTAGTATTACAGAGGTAAGTTATAACAGTCGCCTAACCC
o-YQ309	AATTGGGTTAGGCGACTGTTATAACTTACCTCTGTAATACTAGTGATACCTTAATTAAGGCACAGTCAATGAAGTTTGAAAACCAATAAGAGCTCGGTAAACCT
o-YQ313	ATACTTGATCTAGAAATATTCGCGAGACCGCGGGCAGCTCTGGCCCGTGTCTC
o-YQ314	TAAGTAGACTCGAGGGTTAGGCGACTGTTATAACTTACCTCTGTAATACTAGTGATACCGTCTGACGCTCAGTGGAACGACG
o-YQ321	TAAGTAGATCTAGAGGTACGCGTATTAATTGCGTTGCGCT
o-YQ322	ATACTTGACTCGAGAGTTACGCGTCGTTCCACTGAGC
o-YQ331	CTAGAATTACCCTGTTATCCCTAGGTTTACCGAGCTCTTATTGGTTTTCAAACTTCATTGACTGTGCCGGCCGG
o-YQ332	CCGGCACAGTCAATGAAGTTTGAAAACCAATAAGAGCTCGGTAAACCTAGGGATAACAGGGTAATT
o-YQ333	TCGACGGTGCGTTTTTATGCTTGTAGTATTGTATAATGTTTTTAAGATCCTAGGGATAACAGGGTAATC
o-YQ334	GGCCGATTACCCTGTTATCCCTAGGATCTTAAAAACATTATACAATACTACAAGCATAAAAACGCACCG
o-YQ335	CTAGAGGTCTAATACCCAATCTCTCGTCTTATCCAGATGTTTTATACGCCC
o-YQ336	AATTGGGCGTATAAAACATCTGGATAAGACGAGAGATTGGGTATTAGACCT
o-YQ337	CTAGAGGTGAATTCCCTTATGTGAGTGTAAAAGGCAGGCGAGTTTGTCCCC
o-YQ338	AATTGGGGACAAACTCGCCTGCCTTTTACACTCACATAAGGGAATTCACCT
o-YQ339	CTAGAGGTTGCTTGCAAAAGCAGTAATTGGAAAGCACTCTCAAAGAATCCC
o-YQ340	AATTGGGATTCTTTGAGAGTGCTTTCCAATTACTGCTTTTGCAAGCAACCT
o-YQ341	CTAGAGGTAGATAAGTTGATTTAGCCATAAAATATTGTTTCCGTGACCCCC
o-YQ342	AATTGGGGGTCACGGAAACAATATTTTATGGCTAAATCAACTTATCTACCT
o-YQ349	CTAGAGGTTCTGAGTCACGGCTTCATTGGCATTCCGTACAACGAACGTCCC
o-YQ350	AATTGGGACGTTCGTTGTACGGAATGCCAATGAAGCCGTGACTCAGAACCT
o-YQ351	CTAGAGGTCCTCAGAGAGCCTATAGCGGTAAAACAACACCATGCATCCCCC
o-YQ352	AATTGGGGGATGCATGGTGTTGTTTTACCGCTATAGGCTCTCTGAGGACCT
o-YQ353	AATTGAGGGGATAACGCAGGAAAGAA
o-YQ354	CATGTTCTTTCCTGCGTTATCCCCTC
pZX_1	GGCCTTGGCGCGCCGCTAGCACATGTAAGCTTCGAATTCGGATCCAGC
pZX_2	GGCCGCTGGATCCGAATTCGAAGCTTACATGTGCTAGCGGCGCGCCAA
